# Identifying environmental risk factors for human neural tube defects before and after folic acid supplementation

**DOI:** 10.1186/1471-2458-9-391

**Published:** 2009-10-16

**Authors:** Yilan Liao, Jinfeng Wang, Xinhu Li, Yaoqin Guo, Xiaoying Zheng

**Affiliations:** 1 Institute of Geographical Sciences and Nature Resources Research, Chinese Academy of Sciences, Beijing100101, PR China; 2 Institute of Urban Environment, Chinese Academy of Sciences, Xiamen361003, PR China; 3 Institute of Population Research, Peking University, Beijing100871, PR China

## Abstract

**Background:**

Birth defects are a major cause of infant mortality and disability in many parts of the world. Neural tube defects (NTDs) are one of the most common types of birth defects. In 2001, the Chinese population and family planning commission initiated a national intervention program for the prevention of birth defects. A key step in the program was the introduction of folic acid supplementation. Of interest in the present study was to determine whether folic acid supplementation has the same protective effect on NTDs under various geographical and socioeconomic conditions within the Chinese population and the nature in which the influence of environmental factors varied after folic acid supplementation.

**Methods:**

In this study, Heshun was selected as the region of interest as a surrogate for helping to answer some of the questions raised in this study on the impact of the intervention program. Spatial filtering in combination with GIS software was used to detect annual potential clusters from 1998 to 2005 in Heshun, and Kruskal-wallis test and multivariate regression were applied to identify the environmental risk factors for NTDs among various regions.

**Results:**

In 1998, a significant (p < 0.100) NTDs cluster was detected in the west of Heshun. After folic acid supplementation, the significant clusters gradually moved from west to east. However, during the study period, most of the clusters appeared in the middle region of Heshun where more than 95 percent of the coal mines of Heshun are located. For the analysis, buffer regions of the coal mine zone were built in a GIS environment. It was found that the correlations between environmental risk factors and NTDs vary among the buffer regions.

**Conclusion:**

This suggests that the government needs to adapt the intervention measures according to local conditions. More attention needs to be paid to the poor and to people living in areas near coal mines.

## Background

Birth defects, as defined by the March of Dimes Birth Defects Foundation, refer to any anomaly, functional or structural, that presents in infancy or later in life and is induced by events preceding birth, whether inherited, or acquired. Varying from minor cosmetic irregularities to life-threatening disorders, birth defects are the major cause of infant mortality and a leading cause of disability [1]. NTDs (neural-tube birth defects) are one of the most common forms of birth defects, often occurring between the third and fourth weeks of gestational age. They result in structural defects that occur anywhere along the neuroaxis from the developing brain to the sacrum and often result in the exposure of neural tissue [2].

Birth defects have a substantial public health impact on mortality, morbidity, disability, and to the cost of health care provision. Fortunately, they can often be prevented and early intervention is an important component in the minimization of their consequences. However, for the vast majority of birth defects, the etiology is still unknown.

An environmental cause is any non-genetic factor that increases the risk of a birth defect for the exposed individual [3]. Such factors include fetal infection [4], maternal illness [5], nutritional deficiencies [6,7], drug ingestion [8], chemical exposures [9], air pollution [10], radiation [11], etc. Different birth defects may be caused by different risk factors. In this study, we have limited our research to NTDs.

In recent years, many countries have made efforts to reduce the incidence of birth defects. China has the largest population in the world, and also has the highest incidence of birth defects, with the highest levels compared with the national average being observed in the Shanxi Province [12]. Shanxi also has the highest rate of NTDs in the world. In 1999, the State Family Planning Commission launched the Birth Defect Intervention Project across the country. The four counties of Pingyao, Heshun, Zhongyang and Pingding in Shanxi were designated as the pilot regions for the project carried out in 2001. The intervention work mainly involves dynamic monitoring before and after pregnancy and again following childbirth, and as a result is referred to as a three-tiered intervention. The first-tier intervention service covers newlywed couples and couples who were entitled to have a second child. Before pregnancy, couples were offered counseling and advised to take supplements, including folic acid and iodine. The second-tier intervention covered all pregnant women. In middle and west China, women in some experimental counties took a vitamin supplement containing 0.4 mg folic acid everyday from 3 months before pregnancy to 3 months after pregnancy. However, a 4-mg folic acid supplement daily was advised for women with a previous pregnancy involving a neural-tube defect and women who have taken antiepileptic drugs before. The third-tier intervention covered the effective follow-up treatment of all children who were already affected by birth defects. In this national intervention project, the main measure taken to reduce the risk of NTDs was to counsel women of child-bearing age to take folic acid supplements [12].

**Figure 1:**
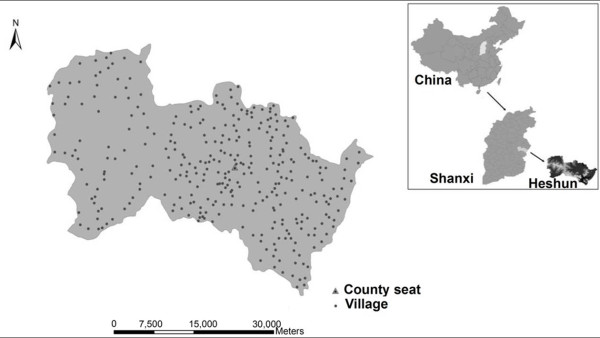
**Location of Heshun**.

Current research suggests that the periconceptional nutritional supplementation of maternal diets with multivitamins, in particular folic acid, is associated with a diminished prevalence and recurrence of NTDs [6]. In the present study we were interested in determining whether folic acid supplementation has the same protective effects on NTDs under various geographic and socioeconomic conditions and whether the influence of environmental factors vary after folic acid supplementation. In this study, we were interested in using the results of the intervention program to provide useful knowledge on the variation of environmental risk factors before and after intervention. The experimental area, the Heshun County, lies in the Tai Hang Mountain Region, at 37°03'E and 113°05'N (see Figure 1). It consists of 326 administrative villages, and has an area of 2,250 km^2^. Most of the people in this county are farmers and their living environments seldom change. Furthermore, there have been no large-scale movements of people in the history of this region. Heshun is national-level poverty-stricken county and local economy is comparatively undeveloped. The quality of life of people living in remote mountainous areas is poor. Daily foods which people there consume include corn, potatoes, beans, tomatoes, fermented vegetables and so on. The inherited and congenital causes of birth defects are similar among the people in this region, and these factors explain only a small fraction of all of the NTDs cases seen. Most types of birth defects designated by the WHO can be found in Heshun, and NTDs predominate [13]. There were 187 NTDs cases reported during the 1998-2005 period in the study region. Correspondingly, there were 7880 births in 1998-2005. During the course of intervention, folic acid supplements readily were available to all women in Heshun. Especially, women who had a previous NTDs-affected pregnancy took forceval capsules which contain a combination of 24 vitamins, minerals and trace elements.

## Methods

### Materials

For this analysis, we included all live and still births occurring in Heshun from January 1, 1998 to December 31, 2005, born to women at the hospital or at home, and who were residents of the county during that time period. We also included all therapeutic abortions in residents in that area where the estimated date of delivery fell in the time period of interest. All NTDs cases, regardless of pregnancy outcome, were verified by doctors in the hospitals. Records of NTDs cases were collected from the local family planning department. The NTDs in the study included anencephaly, spina bifida, encephalocele, and holoprosencephaly, among others. In addition, the Ministry of Science and Technology of the People's Republic of China provided ethics approval for our study.

The local planning department declined to provide identifiers to link substantiated NTDs cases to births, so we were unable to conduct the study at an individual level; instead, we conducted an ecological study. That is, we examined NTDs cases in relation to the environmental characteristics of the villages in which their mothers lived. There were 326 villages and one town in the study area. Since the main object of this study was to find the changes in the relationships between environmental risk factors and NTDs, the town was not included as the environmental factors there are somewhat complex. In addition, birth defect registers in the town were not included.

In the study, the environmental factors of various villages were classified into socioeconomic and geographical factors. The socioeconomic factors reported useful information on medical conditions (the number of doctors), the per-capita incomes (per-capita net incomes), the agricultural chemical exposures (the use of fertilizers and pesticides), and the crop yields (vegetable and fruit productions) of every village during the 1998-2005 period. All socioeconomic data were provided by the Heshun statistical bureau. The geographical factors included elevation, gradient, vegetations coverage (normalized difference vegetation index), access condition (distance to main roads), water shortage condition (distance to rivers) and the geological background (distance to faults) of the villages. The normalized difference vegetation index (NDVI) is an index that provides a standardized method of comparing vegetation greenness between satellite images and can be used as an indicator of relative biomass and greenness. The source of the NDVI dataset used in the study is the VITO (Flemish Inst. Technological Research, Belgium), . In addition, Li et al. [14] found that the occurrence ratio of NTDs in Heshun had a significant negative correlation with increasing-distance from faults. So we chose the distance between villages and faults as an important influence factor.

Geographic Information System (GIS) is a computer package used to store, manage, analyze, and map geographical data. It plays a significant role in data processing and has insuperable advantages over traditional methods. GIS allows the addition of relevant layers which can be used for analyzing the spatial relationships among selected factors. Also, it offers database capabilities that can handle attributes data effectively. Attribute calculations are simple and relatively accurate. We used ARCGIS 9.0i as the GIS platform to locate the 326 villages and to quantify the selected factors.

**Figure 2:**
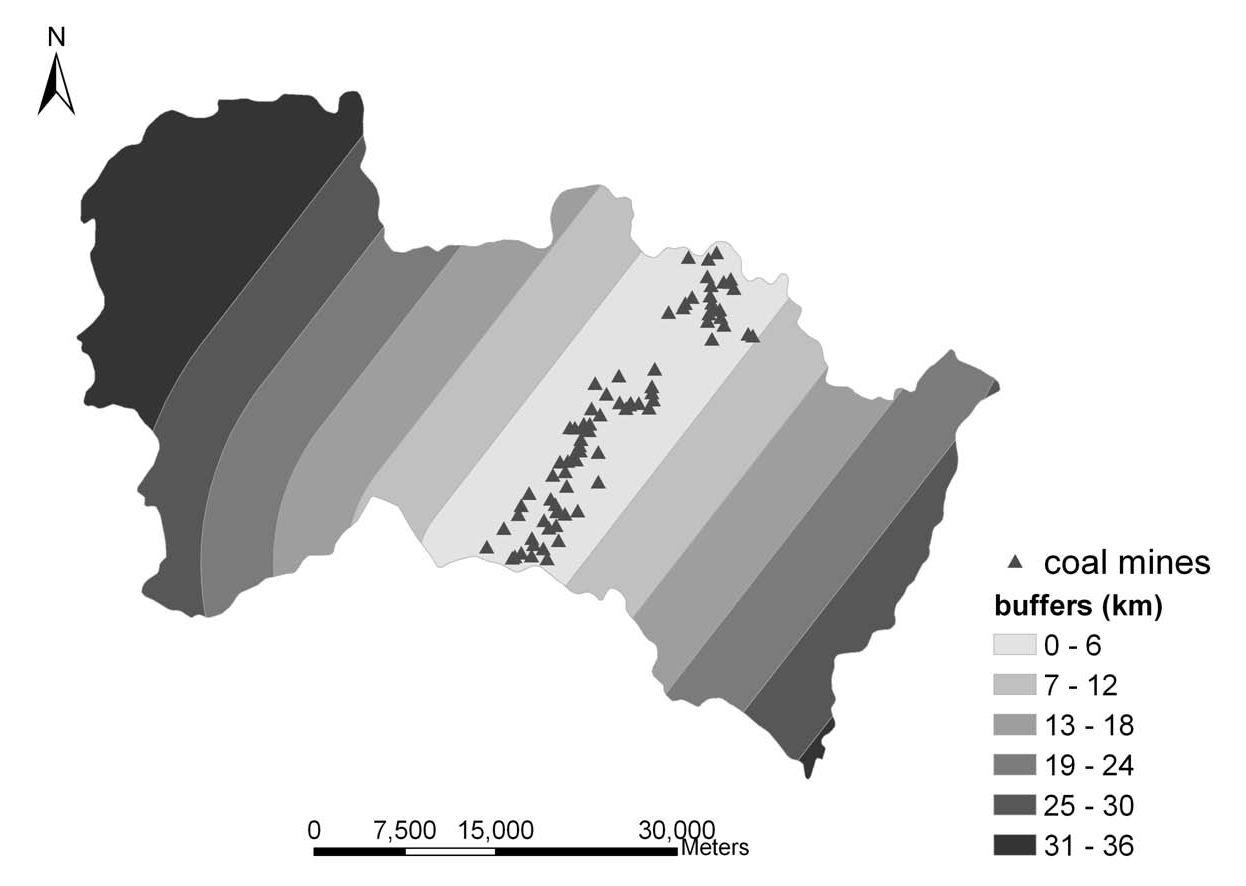
**Distribution of coal mines and their buffer regions**.

For examining the protective effects of folic acid supplementation under various geographic and socioeconomic conditions, the first thing that needed to be carried out was regional division. As is well known, Shanxi is a main base of coal production in China. Was there a correlation between the coal mine distribution and an adverse health risk? Was there a significant increasing effect of folic acid supplementation with increasing distance from the coal mine? The buffer regions of coal mine zones were built into the GIS environment, with buffer distances of 6, 12, 18, 24, 30, or 42 km. As a result, the study region was divided into six zones with buffer distance ranges of 0-6, 7-12, 13-18, 19-24, 25-30, and 31-42 km respectively. Figure 2 illustrates the results of this process.

We assumed that the pilot work of the intervention project began to show effects by 2002. Because NTDs are rare, the NTDs cases reported during the 1998-2001 period and during the 2002-2005 period were combined for the purpose of calculating the occurrence ratios before and after intervention. As a result of the Birth Defect Intervention Project, the rate of NTDs in Heshun reduced from 261.7 per 10,000 births to 215.5 per 10,000 births during the 1998-2005 period.

### Spatial filtering method

The spatial analysis of birth-related morbidity provides a way of identifying inadequate health care access as well as potential environmental and behavioral causal factors. Cluster analysis is frequently used to identify an unusually high occurrence of morbidity that is clustered in space and time [15]. The spatial filtering methodology employs non-parametric statistical techniques as an exploratory spatial data analysis tool. It can provide estimates for the unknown risk or relative risk without making distributional assumptions. The idea behind this technique is to create a series of circular moving windows or disks centered on point locations on a regular grid that covers the study area. For each of the disks, the rate is computed as the crude rate of events to population, or, alternatively, as the ratio of cases to controls. The resulting values at the grid point are then represented in smooth form by means of contour plots, a three-dimensional surface, or similar techniques [16]. This method has been used previously to study clusters of congenital anomalies, infant mortality, and other forms of birth morbidity [15-17]. In the study, we assumed that the probability of a case resulting in a NTDs was equal to the proportion of all births in the region that resulted in NTDs. A 'smoothed' probability map could be drawn where the significance levels of high rates of NTDs by percentage for each individual circle was calculated and mapped in isarithmic form.

### Statistical analysis

The data were statistically analyzed using SPSS Win 13.0 using a 95% level of significance. Kruskal-Wallis test is a nonparametric procedure that can be used to compare more than two populations in completely randomized design. So Kruskal-wallis test was used to compare the average rates of NTDs in the various buffer regions of coal mines. A linear regression analysis was used to investigate the relationship between environmental factors and NTDs. A bivariate correlation analysis was then applied to explore the relationship among different factors. Villages in which the number of births over a five-year period was less than 5 were excluded in the analysis to ensure the reliability of the conclusions. In addition, the crude ORs and 95% CI was applied to explore the association between residences near coal mines and risk of NTDs.

## Results

### Spatial analyses

By matching the addresses of the birth records to village points, we were able to compute the NTDs rate for each location on a grid which covers the entire Heshun County area at a resolution of approximately 4.8 km. In order to be able to make general conclusions about the results of spatial filtering, we used multiple filter sizes such as 1.6, 2.4, 3.2, and 4 km. Based on the cases and births data in 1998, we conducted a sensitivity analysis using differing spatial filter sizes. The result (seen from Table 1) showed that the 4 km size appeared to provide the optimal distance for this study. Progressively larger spatial filtering of data removes local spatial variability [15], which eventually produces an approximately uniform pattern of NTDs.

**Table 1:** The detecting results of different spatial filter sizes

**filter sizes**	**the number of clusters**	**the number of grids included in clusters**
1.6 km	3	3
2.4 km	2	4
3.2 km	2	4
4 km	2	9

The approximately 4.8 km distance interval between grid intersections resulted in 204 grid points in Heshun. Meaningful NTDs rates were estimated for the grid points that had at least 5 births within a 4.8 km vicinity. The spatial filter circle surrounding each of these points is the area from which an estimate of the NTDs rates is made. We counted the normal births and NTDs within the circle and assigned the observed rate to that location. When we repeated this for a grid of such estimates, we were able to interpolate the NTDs rates as a continuous spatial distribution. Neighboring grid points share circular patterns that overlap, thereby sharing observations. Isarithmic maps with a constant range of values were constructed in GIS after the estimated rates were assigned to grid points.

**Figure 3:**
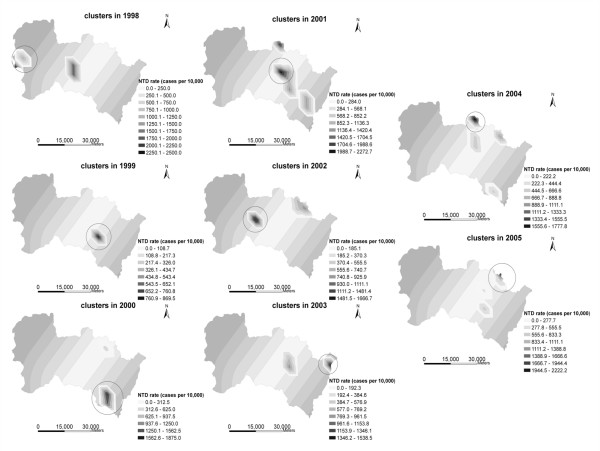
**The NTDs clusters of Heshun during the 1998-2005 period**.

For each village, we generated a random number from a uniform distribution in the range of 1 through 999. For each of the 204 grid-point locations, 999 Monte Carlo simulations were made. The simulated and real rates were then rank-ordered. The percent of the simulated rates at each grid location that were less than the observed rate for the same grid location were computed and the levels of statistical significance were portrayed as isolines. Because testing the rates against 999 simulations is a form of exploratory spatial analysis, the methods for representing the results are discretionary, and the investigator can adjust the results based on the level of significance. Figure 3 illustrates the NTDs clusters for Heshun during the 1998-2005 period.

### Kruskal-wallis test

As seen in Figure 3, clusters of NTDs were often seen in the buffer regions of 0-6 and 7-12 km during the study period. Although the government had initiated a birth defects intervention project two years previously, the hot spots for the NTDs continued to appear in these buffer regions into the years 2004 and 2005. Correspondingly, the buffer region of 31-42 km had only one cluster of NTDs in 1998.

**Figure 4:**
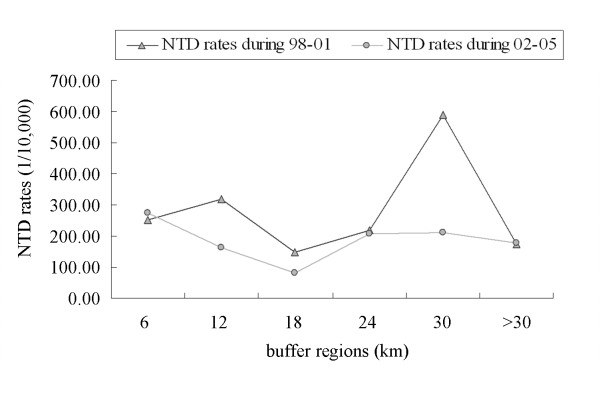
**The average NTDs rates of the buffer regions before and after folic acid supplementation**.

Does this phenomenon mean that the pollution from coal mines may affect the incidence of NTDs in Heshun? Does this effect gradually reduce with increasing distance from coal mines? The average NTDs rates for every buffer region before and after folic acid supplementation were calculated and illustrated in Figure 4. During the 1998-2001 period, the average NTDs rates in the buffer range of 0-24 km followed the expected distribution as a whole. However, the highest value appeared in the buffer regions of 25-30 km because of the influence of other factors. After folic acid supplementation, the trend in the form of the "M"-shaped curve changed, and the average NTDs rates in many regions decreased significantly. The region near the coal mines maintained their high prevalence of NTDs.

Were these differences among various buffer regions able to be eliminated by the folic acid supplementation? The results of Kruskal-wallis test (seen in Table 2) showed that the NTDs rates for the 2002-2005 period differed significantly among all of the buffer regions. Nevertheless, the same phenomena did not appear during 1998-2001.

**Table 2:** Kruskal-Wallis test results of the average NTDs rates of the buffer regions before and after folic acid supplementation

**period**	**Chi-Square**	**Degrees of Freedom**	**P**
1998-2001	4.497	5	0.480
2002-2005	14.224	5	0.014

### Multivariate regression analysis

The region within 6 km of a coal mine was found to be the one with the highest prevalence of NTDs in all cases. The pollution levels associated with coal mines is believed to be the reason for the high levels of NTDs observed there. In the range of 7-12 km, the incidence of NTDs decreased greatly through folic acid supplementation. The multivariate regression analysis (seen in Table 3) revealed that the number of doctors was directly proportional to NTDs incidence before folic acid supplementation (*r *= 0.511, *p *= 0.001). The villages with more doctors tended to have more NTDs. After 2001, fruit production (*r *= -0.479, *p *= 0.028) and distance to a fault (*r *= -0.531, *p *= 0.013) were found to negatively correlate with NTDs in that area. The distance of the births became closer to factories, the prevalence of NTDs decreased (*r *= 0.380, *p *= 0.026). The 13-18 km range was always the one found to have the lowest rate. The risk factors there changed from elevation (*r *= 0.437, *p *= 0.026) to the number of doctors (*r *= -0.717, *p *= 0.000) and distance to the roads (*r *= -0.323, *p *= 0.016) and rivers (*r *= 0.440, *p *= 0.002) through folic acid supplementation. The effect of folic acid supplementation was the most obvious in the range of 25-30 km. A statistically significant positive correlation was found between NTDs and gradient (*r *= 0.602, *p *= 0.004) there during 2002 - 2005. However, the NTDs rates in the remaining buffer regions remained essentially unchanged with the introduction of folic acid supplementation. The selected environmental risk therefore seems not to be correlated with the NTDs there.

**Table 3:** Multivariate regression analyses of the association between environmental factors and NTDs as the dependent variable

**Model (R^2^) and Independent Variables**	**Standardized Coefficients**	**t**	**P**	**Std. Error**
Rates for the 1998-2001 period in the buffer region of 7-12 km (0.415)				
doctor	0.511	3.683	0.001	7.096
Rates for the 2002-2005 period in the buffer region of 7-12 km (0.242)				
fruit	-0.479	-2.298	0.028	15.020
pollution	0.380	2.337	0.026	0.003
Distance to faults	-0.531	-2.617	0.013	0.003
Rates for the 1998-2001 period in the buffer region of 13-18 km (0.191)				
elevation	0.437	2.380	0.026	0.146
Rates for the 2002-2005 period in the buffer region of 13-18 km (0.711)				
doctor	-0.717	-5.848	0.000	8.693
distance to roads	0.440	3.640	0.002	0.004
distance to rivers	-0.323	-2.618	0.016	0.002
Rates for the 1998-2001 period in the buffer region of 25-30 km (0.486)				
fertilizer	0.540	3.177	0.005	0.640
elevation	0.392	2.308	0.033	0.153
Rates for the 2002-2005 period in the buffer region of 25-30 km (0.362)				
gradient	0.602	3.283	0.004	1.836

## Discussion

The study suggests that the effect of folic acid supplementation varied among the different subzones in Heshun. The NTDs rates in the buffer regions of 7-12, 13-18, and 25-30 km decreased significantly due to folic acid supplementation. However, folic acid supplementation did not play a role in reducing the rate in the buffer regions of 0-6, 19-24, and 31-42 km. In fact, the rate for the sub-region near the coal mines zone was actually found to increase. The environmental risk factors in these sub-regions are not the same. It is significant that we were able to identify environmental correlates of NTDs among the sub-regions.

The buffer region of 0-6 km is the area close to the coal mines. Spatial filtering detected clusters of NTDs in this area almost every year. Infants there had a significantly higher risk of NTDs compared with those living in other areas throughout the whole of the study period (OR = 1.347; 95% CI = 1.004-1.808). So, in addition to the three-tiered intervention program, the government should strengthen its local environmental governance.

Many of the villages in the buffer region of 7-12 km are located at high altitudes and located at significant distances from rivers. This area is an economically deprived area of Heshun and lacks agricultural production materials such as fertilizer and pesticide. Before intervention, there was a poor state of health amongst the population and many villages had no doctor. People generally saw a doctor at the rural clinics of the neighboring villages. Some of the records of NTDs cases were collected from clinics and hospitals, so the number of doctors was in direct proportion to the NTDs incidence during the 1998-2001 period. With the implementation of interventions, the number of rural doctors increased substantially. The production of fruit, distance to factories and to faults were correlated with NTDs in these areas. It was unexpected that the number of NTDs was found to increase with increasing distance from factories. Taking the spatial interaction into account, we qualified the environmental factors of neighboring villages within a specified distance and then explored the relationships between them and the distance to factories. The number of doctors (*r *= -0.364, *p *= 0.029), NDVI (*r *= -0.420, *p *= 0.011), vegetable production (*r *= -0.502, *p *= 0.002) and per capita net income (*r *= -0.420, *p *= 0.011) were all negatively correlated with this variable. So the factor variable 'distance to factories', could be taken as a composite indicator of the peripheral environment. Faults indicate intensive geological structural movement. And the concentration of radon in soil, water, and air near fault zones were found to be far higher than away from faults [14]. However, it needs to approve in the future whether a fetus in utero is at particular risk of damage from radiation of radon. In previous work, the higher rates of certain types of NTDs, such as anencephaly and spina bifida, were found to be associated with living at lower altitude [18]. However, in our study, the elevation was found to be in direct proportion to the NTDs for the 1998-2001 period in the 13-18 km buffer region. Similarly, we tried to understand the relationship between the neighboring environmental characters of villages where NTDs cases occurred and their elevations. In the neighborhood of villages with higher altitudes, the number of rural doctors (*r *= -0.473, *p *= 0.015), the use of chemical fertilizers (*r *= -0.821, *p *= 0.000), the fruit yield (*r *= -0.645, *p *= 0.000), and the per capita net income (*r *= -0.535, *p *= 0.005) of neighbors were relatively small. The difficult living environments made it more likely for the infants in these villages to contract NTDs. This is different from the 7-12 km buffer region. The 13-18 km buffer region was covered by dense vegetation, and the vegetable yield there continued to increase during the 2002-2005 period.

Nevertheless, limited medical access, and conditions of water shortage were the main handicaps in reducing NTDs prevalence in these areas.

Although no selected environmental risk factors related directly to NTDs in the 19-24 km buffer, we found that the use of fertilizers and pesticides was the highest in the county. Moreover, the production of fruit in this buffer was far less than the one in the 13-18 km buffer. This may be used to explain why the rate curve of NTDs there suddenly became higher.

The 25-30 km buffer region used more fertilizers than the other areas before 2002. The multivariate regression analysis found that the use of fertilizers was directly related to NTDs rates there at that time. Fertilizers are organic or inorganic substances, either natural or synthetic, used to supply elements (such as nitrogen, phosphate and potash) essential for plant growth. However, over application of nitrogen fertilizers and loss of phosphate fertilizer due to soil erosion may result in some undesirable environmental problems such as increase of nitrate in water and eutrophication of water bodies [19]. Croen et al. [20] found that exposure to nitrate in drinking water at high concentrations was associated with increased risk for anencephaly. Moreover, the surrounding environment of the villages with higher elevations was also badly affected. So elevation became the other factor which associated with NTDs in this region. After 2001, this sub-region underwent a period of strong economic growth and the production of vegetables increased year by year. Infants in the villages with a higher slope had a higher risk of NTDs. From our investigation, it was found that most of these villages are in mountainous regions or regions lacking in water. There is significant scope to further reduce NTDs in this sub-zone by improving the quality of life of these residents.

However, we have not found any selected influence factors of NTDs in the buffer region of 31-42 km yet. So we cannot give any explanation for no change in NTDs there and attempt to deal with this problem in further work.

## Conclusion

Although we found that folic acid supplementation had different effects on the various buffer regions, there are some limitations associated with our choice of methodology. A concern is that there may have been a significant amount of underreporting in this area, as some pregnant women would have chosen to have home births rather than hospital births. Therefore, we were limited to the use of data from hospital records and investigations undertaken in the villages. Also, some of the women may have relocated during their pregnancies, so there could be a migration bias in risk factor identification. In addition, our study is limited to a ecological analysis since we were unable to obtain any individual information otherwise required. The measures of environmental exposure are limited. Causes of individual NTDs cases cannot be identified, and the maternal characteristics of each NTDs is unknown.

Given that the causes of NTDs vary among sub-regions, it is sensible to adopt different intervention methodologies for each region. More attention should be paid to areas near coal mines. Needless-to-say, there is also an urgent need to try to improve the quality of life of people living in remote mountainous areas. Although in this study we only had the opportunity to investigate the effects of the birth defects intervention project in Heshun, a rural region in the north of China, the analysis methodology may provide some good ideas to Chinese government to improve treatment policies and intervention measures for birth defect in the future.

## Competing interests

There are non-financial competing interests (political, personal, religious, ideological, academic, intellectual, commercial or any other) to declare in relation to this manuscript.

## Authors' contributions

YL carried out all analysis and drafted the manuscript. XH participated in the design of the study and provided materials to explain the results of the study. YQ took part in collecting data. JF and XY conceived of the study, and participated in its design and coordination and helped to draft the manuscript. All authors read and approved the final manuscript.

## Pre-publication history

The pre-publication history for this paper can be accessed here:


